# Role of Intraoperative Frozen Section Assessment of Proximal Bile Duct Margins and the Impact of Additional Re-Resection in Perihilar Cholangiocarcinomas

**DOI:** 10.1007/s13193-024-01874-5

**Published:** 2024-01-09

**Authors:** Gurudutt P. Varty, Mahesh Goel, Kunal Nandy, Kedar Deodhar, Tanvi Shah, Shraddha Patkar

**Affiliations:** 1 Department of Gastrointestinal and Hepatobiliary Surgery, Department of Surgical Oncology, Tata Memorial Hospital, Homi Bhabha National Institute, Mumbai, Maharashtra India; 2 Department of Surgical Pathology, Tata Memorial Hospital, Homi Bhabha National Institute, Mumbai, Maharashtra India; 3 Department of Surgical Oncology, Tata Memorial Hospital, Homi Bhabha National Institute, Mumbai, Maharashtra India; 4 Homi Bhabha Block, Tata Memorial Hospital, Ernest Borges Road, Room Number 1204, 12th floor, Parel East, Mumbai, 400012 India

**Keywords:** Perihilar cholangiocarcinoma, Frozen section, Proximal margin positivity, Resection of proximal margins

## Abstract

Intraoperative frozen section (FS) analysis to assess the bile duct margin status is commonly used to assess the completeness of resection during surgery for perihilar cholangiocarcinoma (pCCA) resection. However, the impact of additional re-section on the long-term outcome after obtaining an initial positive margin remains unclear. Patients diagnosed as pCCA on preoperative imaging and subjected to curative intent surgery from May 2013 to June 2021 with a minimum follow-up of 2 years were included. Intraoperative FS analysis of the proximal bile duct margin was performed in all patients. A positive margin was defined by the presence of invasive cancer. Out of the 62 patients with a preoperative diagnosis of pCCA on imaging, 35 patients were included for final analyses after excluding patients with inoperable disease (on staging laparoscopy or local exploration) and other/benign pathology on the final histopathology report. Out of the 35 patients, patients with postoperative 90-day mortality were excluded from the final survival analysis. FS analysis revealed an initial positive margin in 10 (28.5%) patients. Among 10 patients who underwent re-resection to achieve negative proximal margins, only 5 patients achieved a negative margin (secondary R0). An initial positive margin was associated with poor long-term outcomes. Median disease-free survival (DFS) and overall survival (OS) were 16 and 19.6 months for patients with an initial positive margin, but 36 and 58.2 months for patients with an initial negative margin, respectively (*p* = 0.012). The median DFS and OS were significantly lower for those with secondary R0 as compared to primary R0 (16 vs. 36 months for DFS, *p* = 0.117 and 19.6 vs. 58.2 months for OS, *p* = 0.027, respectively). An intraoperative FS positive proximal hepatic duct margin dictates poor long-term outcomes for patients with resectable pCCA. Additional resection has a questionable benefit on survival, when a secondary negative margin is achieved.

## Introduction

Perihilar cholangiocarcinoma (pCCA) is an uncommon malignancy arising from the biliary epithelium at the hilum of the liver, accounting for approximately 50% of all cholangiocarcinomas, and around 3% of all gastrointestinal malignancies. [1] Surgical resection is the only potentially curative treatment modality available for long-term survival [2–4]. In order to attain the ultimate goal of potential ‘cure’, achieving a complete resection with microscopically cancer-free proximal and distal margins is an essential prerequisite [5, 6]. To maximise the chance of cancer-free margins, hepatectomy (hemihepatectomy or extended) with en bloc extrahepatic bile duct resection and a Roux-en-Y hepaticojejunostomy is advocated as a standard operation for non-metastatic and resectable pCCA [7]. Despite this radical extirpative surgery, the incidence of incomplete (R1) resection with a positive margin remains high (10–72%) [8–11].

Intraoperative frozen section (FS) is routinely utilised at many centres to assess the margin status and adequacy of resection at the hilar and intraparenchymal resection planes (proximal) and common bile duct (CBD) margins at the duodenal side (distal). When the proximal margin is positive for malignant cells, additional resection of hepatic duct is considered if technically feasible. Similarly, if distal margin is positive for cancer, additional resection of the distal bile duct is attempted, or rarely, a pancreaticoduodenectomy may be considered. Several studies have investigated the value of additional resection after positive FS and its influence on overall survival (OS) [10–15].

However, the feasibility and efficacy of revising the proximal margins based on FS do remain controversial because of technical limitations, postoperative morbidity and its true impact on OS. Thus, this study was performed to address whether additional resection of proximal margins after performing an intraoperative FS translates into an improved survival in pCCA patients.

## Methods

### Patient Cohort

**Fig. 1:**
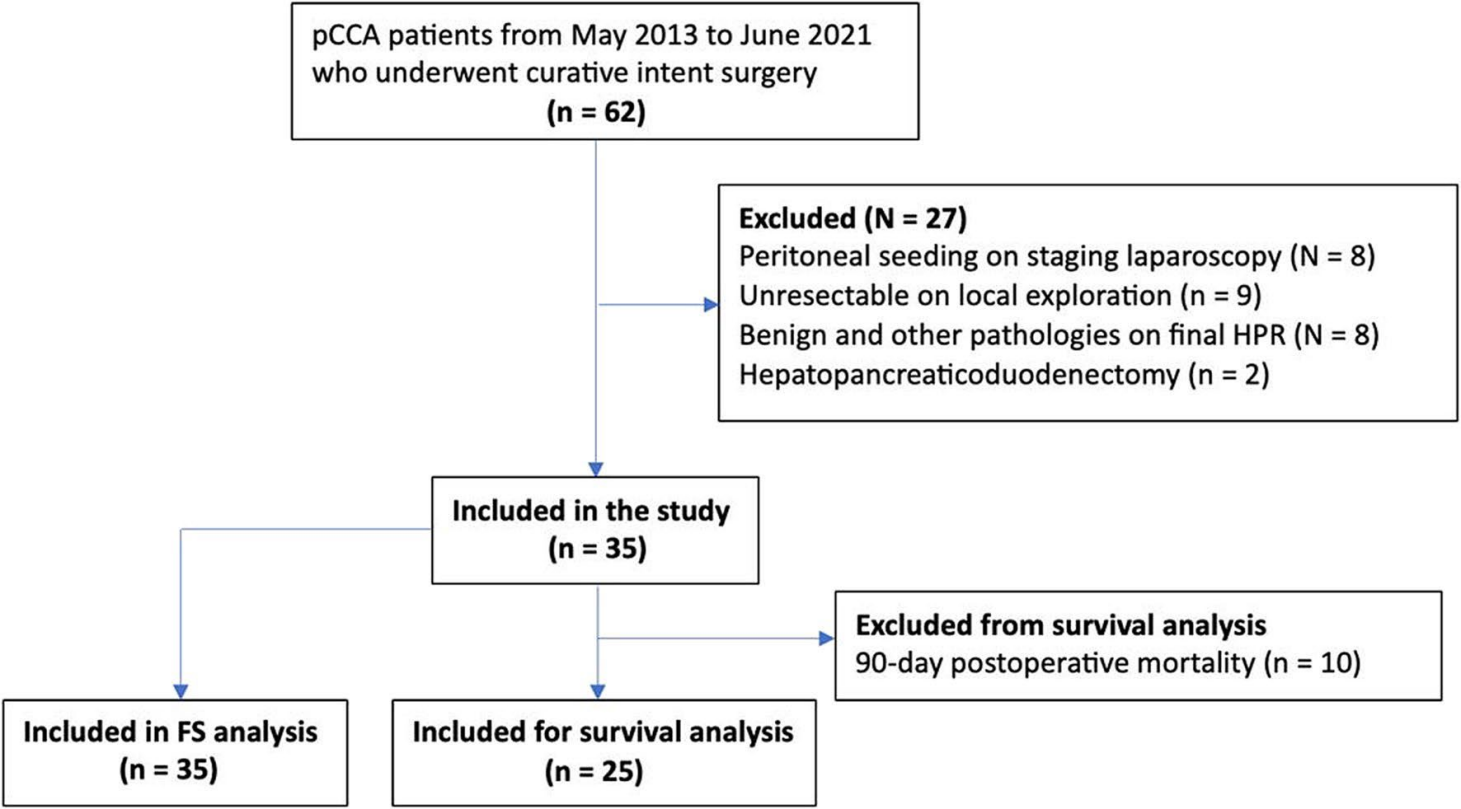
Selection of study population. pCCA, perihilar cholangiocarcinoma; HPR, histopathology report; FS, frozen section

Prospectively, a maintained departmental database of pCCA of a single, high-volume hepatobiliary surgical oncology unit was retrospectively analysed. The study cohort consisted of all consecutive patients who were preoperatively diagnosed as pCCA and underwent curative intent surgery between May 2013 and June 2021. Patients who were deemed to have gross metastatic disease on staging laparoscopy or inoperable on local exploration were excluded. Similarly, patients with other pathologies such as carcinoma gallbladder or benign disease on final histopathology and tumour extension to final distal CBD margins and those with missing data were also excluded (Fig. 1). The clinical and histopathological data were retrieved from the database, supplemented by electronic medical record system.

### Management of Resectable pCCA

All patients were presented at our dedicated weekly ‘Liver clinic’ multidisciplinary team discussion, and the resectability and operative plans were discussed. A triphasic contrast-enhanced computed tomography (CECT) scan was performed for primary staging. Magnetic resonance cholangiopancreatography (MRCP) was done in cases where further information was needed to know the type of block in pCCA as per the Bismuth-Corlette (BC) classification [16]. Positron emission tomography (PET) scan was done to rule out suspicious extrahepatic metastases selectively. Patients who presented with obstructive jaundice routinely underwent percutaneous transhepatic biliary drainage (PTBD) or endoscopic retrograde cholangiopancreatography (ERCP)-guided biliary drainage. As an institutional protocol, PTBD was preferred over ERCP for biliary drainage in order to provide a better assessment of the proximal extent of the disease. The tumour was considered resectable when R0 resection could be performed with at least 35% of the future liver remnant (FLR) left behind. When the anticipated FLR was considered inadequate, patients underwent portal vein embolization (PVE). Contralateral hepatic artery invasion was considered a contraindication to resection whereas portal vein invasion was not, and a portal vein resection with reconstruction was planned. If the tumour extended distally into the CBD and pancreas, hepatopancreaticoduodenectomy was undertaken selectively. However, such cases were excluded from the study to prevent confounding.

### Surgery and Intraoperative Assessment of Margins by FS

Staging laparoscopy was performed routinely in all cases to rule out gross metastatic disease, and after confirming operability after local exploration, patients underwent a formal right/left hepatectomy or an extended right/left hepatectomy along with caudate lobectomy with extrahepatic biliary tract resection and a complete portal lymphadenectomy (stations 8, 12 and 13). Portal vein resection and reconstruction was done when deemed necessary. Once the specimen was delivered, a 2 to 3-mm cuff of the cut end of the proximal bile duct was obtained from the specimen and submitted for FS evaluation with the margin inked. When invasive cancer was confirmed on FS examination, an additional cuff of the proximal bile duct was excised from the remnant liver when considered feasible. However, submission of the re-resected margins for FS was at the surgeon’s discretion. The margin submitted for FS was subsequently revaluated at the final histopathology. Bilioenteric continuity was established by a Roux-en-Y hepaticojejunostomy. R0 was defined as the absence of malignant cells (≥ 1 mm), and R1 was defined as the presence of malignant cells (< 1 mm) at the proximal margins [17, 18]. Other pathological stages were reported according to the 8th edition of the Union for International Cancer Control (UICC) staging system [19].

### Post-Surgical Outcomes and Follow-Up

All post-surgery complications were recorded based on International Study Group of Liver Surgery (ISGLS) criteria and classified as per the Clavien–Dindo classification. Patients were followed up at three monthly intervals or sooner if indicated for the first 2 years. Follow-up evaluation included a clinical assessment, complete blood count, liver function tests, tumour markers (CA-19,9, CEA), chest X-ray and abdominal ultrasound. A CECT of the abdomen and thorax was performed at six monthly intervals for the first 2 years and annually thereafter. When the disease recurrence was suspected, further imaging and/or histological confirmation was sought and documented.

### Statistical Analysis

Disease-free survival (DFS) and OS were measured from the time of resection to the time of detection of first recurrence, and the time of death/last follow-up, respectively. Continuous variables were presented as means ± standard deviation or medians (IQR). Categorical variables were presented as numbers (n) and proportions (%). Student’s *t*-test or the Mann − Whitney U test was used to compare continuous variables. χ2 test or Fisher’s exact test was used to compare categorical variables. Median follow-up was calculated by the reverse Kaplan–Meier method, and survival curves were estimated by the Kaplan–Meier method and compared using the log rank test. Statistical analyses were performed using SPSS software version 27.0 (SPSS, Chicago, IL, USA).

## Results

A total of 62 patients underwent curative intent surgery for pCCA between May 2013 and June 2021. Finally, 35 patients who fulfilled the study criteria were included in the final analysis (Fig. 1). The median age of the entire cohort was 54 (29–75) years with a male preponderance (68.5%). Twenty-eight (80%) patients received preoperative biliary drainage, with preference to PTBD in 21 (60%) patients. BC type > II tumour was seen in 31 (88.5%) patients. Thirty-one patients (88.6%) underwent hemihepatectomy with an en bloc caudate lobe, and 4 (11.4%) patients underwent trisectionectomy. Portal vein resection was necessary in 5 (14.3%) patients. The postoperative morbidity (Clavien–Dindo grade > 3) of the entire population was 25.7% with a median hospital stay of 15 (5–37) days. In terms of tumour characteristics, 23 (65.7%) patients had moderately differentiated adenocarcinoma, with 8 (22.8%) patients having node-positive disease on final histopathology. While 14 patients (40%) were kept on observation post-surgery, 9 (25.7%) patients received adjuvant chemotherapy, and 2 (5.7%) patients received chemotherapy with local radiotherapy. The commonest indication of giving adjuvant chemotherapy was node-positive and margin-positive cancers. Radiotherapy in addition to adjuvant chemotherapy was preferred in margin-positive cancers.

During the follow-up period, 14 (40%) patients had recurrence out of which 11 (31.42%) had distant recurrence. The distribution of preoperative, intraoperative, histopathological and postoperative variables in patients with initial FS negative versus initial FS positive is depicted in Table 1. The occurrence of node positivity on final HPR was found to be higher in initial FS positive patients as compared to initial FS negative (5 patients (50%) vs 3 patients (12%), *p* = 0.016, respectively).

**Table 1:** Distribution of variables in patients with ‘Initial R0’ versus ‘Initial R1’

Variable	Total (*n* = 35)	Initial R0 (*n* = 25)	Initial R1 (*n* = 10)	p value
Patient characteristics				
Age, median (range)	54 (29–75)	53 (29–75)	54.5 (41–72)	0.957
Male, *n* (%)	24 (68.57%)	16 (64%)	8 (80%)	0.357
BMI, mean (SD)	21.60 (3.47)	21.80 (3.51)	21.13 (3.51)	0.615
ASA (> 2), *n* (%)	2 (5.7%)	2 (8%)	0	0.350
Preoperative biliary drainage, *n* (%)	28 (80%)	19 (76%)	9 (90%)	0.627
None	7 (20%)	6 (24%)	1 (10%)	
PTBD	21 (60%)	14 (56%)	7 (70%)	
ERCP	7 (20%)	5 (20%)	2 (20%)	0.183
CEA, median (ng/ml)	3.10 (1–36)	3.74 (2–25)	2.10 (1–36)	0.219
CA 19–9, median (u/ml)	64.33 (2–11,454)	37.63 (2–11,454)	221.8 (2–5910)	
BC classification, *n* (%)				0.594
I	0	0	0	
II	4 (11.4%)	3 (12%)	1 (10%)	
IIIa	15 (42.85%)	9 (36%)	6 (60%)	
IIIb	13 (37.14%)	10 (40%)	3 (30%)	
IV	3 (8.57%)	3 (12%)	0	
Operative variables				
Operative type, *n* (%)				
Hemihepatectomy	31 (88.57%)	21 (84%)	10 (100%)	0.09
Right	15	8 (32%)	7 (70%)	
Left	16	13 (52%)	3 (30%)	
Trisectionectomy	4 (11.42%)	4 (16%)	0	1
Right	2 (5.71%)	2 (8%)		
Left	2 (5.71%)	2 (8%)		
PV resection, *n* (%)	5 (14.28%)	3 (12%)	2 (20%)	0.541
Blood loss, median (ml)	2700 (900–8000)	2500 (900–7600)	2910 (1300–8000)	0.378
Postoperative variables				
Hospital stay, median (range)	15 (6–37)	13 (6–26)	19 (8–37)	0.871
PHBL	12 (34.28)	9 (36%)	3 (30%)	0.735
Morbidity–CD ≥ 3, *n* (%)	9 (25.71%)	7 (28%)	2 (20%)	0.629
90-day mortality, *n* (%)	10 (28.5%)	7 (28%)	3 (30%)	0.906
Tumour pathology				
Differentiation, *n* (%)				
WDAC	5 (14.28%)	5 (20%)	0	0.088
MDAC	23 (65.71%)	17 (68%)	6 (60%)	
PDAC	7 (20%)	3 (12%)	4 (40%)	
Microinvasion, *n* (%)				
LVI	14 (40%)	8 (32%)	6 (60%)	0.127
PNI	24 (68.57%)	15 (60%)	9 (90%)	0.084
pN, *n* (%)				
N0	27 (77.14%)	22 (88%)	5 (50%)	0.016
N1/2	8 (22.85%)	3 (12%)	5 (50%)	
Adjuvant therapy, *n* (%)				
None	14 (40%)	13 (52%)	1 (10%)	0.032
Chemotherapy	9 (25.7)	4 (16%)	5 (50%)	
CTRT	2 (5.7%)	1 (4%)	1 (10%)	
Recurrence, *n* (%)	14 (40%)	8 (32%)	6 (60%)	0.178
Local only	3 (8.5%)	1 (4%)	2 (20%)	0.347
Distant	11 (31.42)	7 (28%)	4 (40%)	

*BMI* body mass index, *ASA* American Society of Anesthesiologists, *PTBD* percutaneous transhepatic biliary drainage, *ERCP* endoscopic retrograde cholangiopancreatography, *BC* Bismuth-Corlette, *PV* portal vein, *CD* Clavien–Dindo, *PHBL* post-hepatectomy bile leak, *WDAC* well-differentiated adenocarcinoma, *MDAC* moderately differentiated adenocarcinoma, *PDAC* poorly differentiated adenocarcinoma, *LVI* lymphovascular invasion, *PNI* perineural invasion, *CTRT* chemotherapy-radiotherapy

### Intraoperative FS Analysis

**Fig. 2:**
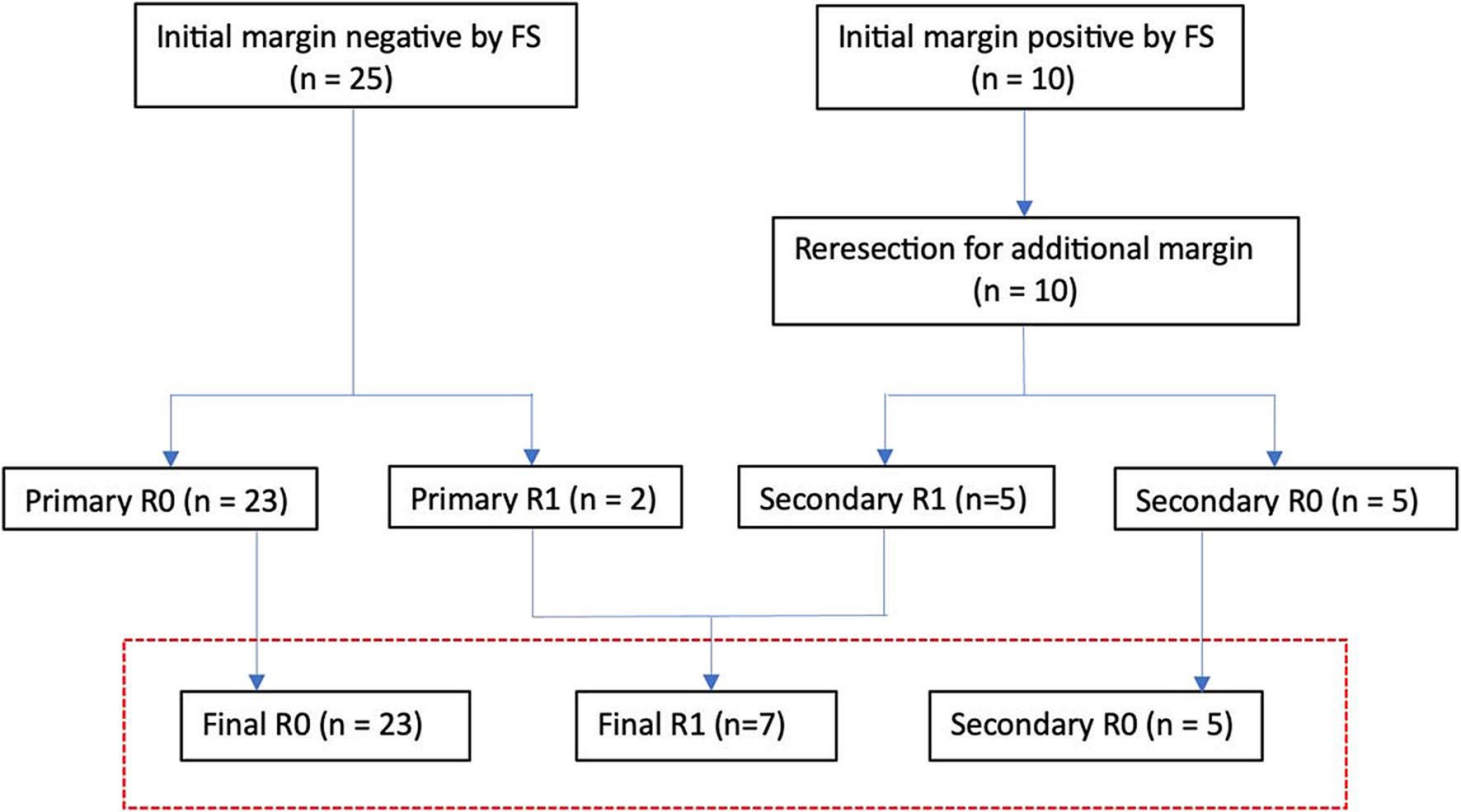
Flow diagram depicting the proximal margin status on frozen section analysis and final histopathology. FS, frozen section

Intraoperative FS analysis was performed in all 35 patients, and 10 (28.57%) were diagnosed as margin-positive on FS analysis. Re-resection of margins was performed in all 10 patients, 5 (14.28%) out of which achieved margin-negative resection on final HPR (secondary R0). Out of the 25 (71.42%) patients which were diagnosed as margin-negative on FS, 23 (65.71%) were margin-negative on final HPR (primary R0). Thus, finally, 23 (65.71%), 7 (20%) and 5 (14.28%) were ‘final R0’, ‘final R1’ and ‘secondary R0’, respectively (Fig. 2). The sensitivity and specificity of intraoperative FS analysis were 71.43% and 100%, respectively (Table 2).

**Table 2:** Accuracy calculation of frozen section analysis in assessing the proximal bile duct margins in resected pCCA

Proximal margin status	Positive on final HPR	Negative on final HPR	Total
Positive on FS	5	0	4
Negative on FS	2	28	31
	R1 = 7	R0 = 28	

Sensitivity: 71.43%; specificity: 100%; positive predictive value: 100%; negative predictive value: 94. 29%

*HPR* histopathology report

### Impact of Initial R1 Margins and Re-Resection on Long-Term Outcomes (Table 3)

**Table 3:** Survival analysis

	Median OS (months)	1-year OS (%)	2-year OS (%)	*p* value	Median DFS (months)	1-year DFS (%)	2-year DFS (%)	*p* value
Intraoperative FS								
Negative	58.2	98.3	51	0.012	36.0	66.7	44.4	0.085
Positive	19.6	51.4	17.1		16.06	38.1	0	
Final margin								
R0	45.79	94.1	52	0.027	17.97	46.3	38.6	0.094
R1	23.45	53.3	0		12.32	30	0	
Final margin								
Primary R0	58.2	92.9	56.3	0.027	36	64.2	47.6	0.117
Secondary R0	19.6	66.7	0		16.06	33.33	0	
R1	23.5	53.4	0		12.32	30	0	

*FS* frozen section, *OS* overall survival, *DFS* disease-free survival

The median follow-up period for the 35 patients included in the study was 26 months. For survival analysis, patients with postoperative 90-day mortality were excluded. The median DFS and OS for the final R0 resection were 17.97 and 45.79 months versus 12.32 and 23.45 months, respectively, for the final R1 resection (*p* = 0.027 for DFS and *p* = 0.094 for OS). The median DFS and OS were 16 and 19.6 months for patients with an initial positive margin on FS, but 36 and 58.2 months for patients with an initial negative margin, respectively (*p* = 0.085 for DFS and *p* = 0.012 for OS).

Although adjuvant therapy was used more in patients who were initial margin-positive on FS (60% vs. 20%, *p* = 0.032), it did not improve their outcomes as compared to those patients who were initial margin-negative on FS. Three patients (2 out of the 5 patients, who achieved secondary R0, died postoperatively) out of the 7 patients who underwent re-resection for an initial positive on FS achieved final R0 on HPR (secondary R0). The 1-year DFS and OS for secondary R0 was 33.3% and 66.7% versus 30% and 53.4% for final R1 resection, respectively, while none of the patients survived at the end of 2 years. The median DFS and OS for patients who achieved primary R0 was higher as compared to those who achieved secondary R0 (36 and 58.2 months vs. 16.06 and 19.06 months, respectively, *p* = 0.027) This suggests that achieving a secondary R0 by re-resecting the proximal margins may or may not improve survival compared to R1 resection, but the survival benefit is definitely less than for the patients who achieved primary R0.

## Discussion

Perihilar cholangiocarcinoma often presents as a stricture-forming lesion, unlike most other tumours which present as discrete masses. This makes assessment of the longitudinal spread of the disease difficult by preoperative imaging. Although a metaanalysis showed that a multidetector CECT has 86% accuracy in determining the proximal extent of the disease spread, microscopic extension is often much greater [20]. In order to address this discrepancy, the intraoperative FS section of ductal margins is widely practised which aims to assess this microscopic spread and offer a chance of re-resection if the initial margin is positive on FS. Presumably, an additional negative ductal margin excision for an initial margin-positive, confirmed on FS, should likely translate to a greater survival [21]. However, initially, positive margins denote tumour transection and potential local periductal and peritoneal dissemination, which may mitigate the benefits of further resection of a positive proximal ductal margin on long-term outcomes [22]. Intraoperative FS, although practised widely throughout the world, has certain issues which need to be addressed before accepting its role beyond doubt.

Reports regarding discrepancy between the intraoperative FS analysis and final HPR report are varied. While some centres report a false negativity rate of 10 to 16% [11, 13, 14], one recent study has reported a false negativity rate as low as 1% [23]. Mantel et al. and Okazaki et al. reported a sensitivity of 68% and 75%, respectively [11, 24]. Our study demonstrates a false negative rate of 28.5% along with a sensitivity of 71.43% for intraoperative FS analysis of proximal margins (Table 2). Various factors for this misdiagnosis on FS possibly include inflammation at the proximal margins secondary to biliary drainage procedures and characteristic growth pattern of longitudinal infiltrative extension along the mucosa and submucosa resulting in sampling errors [11, 23]. The specificity of FS in our study was 100% suggesting that when FS of the margin is negative on initial assessment, one can be relatively sure of the final HPR negative margins (R0).

In our study, 50% of patients who were initially positive on FS got converted to margin-negative resection on final HPR after re-resection (secondary R0) while the other 50% remained R1 resection. It is important to note that re-resection of margins in pCCA may not be as straightforward as in other malignancies (e.g. colon) as a long proximal bile duct stump is seldom left behind during initial resection. The reported success rate of converting a R1 margin to a R0 is modest ranging from 32 to 83% [9, 10, 13–15, 25]. Re-resection of the margins also makes the hepaticojejunostomy technically difficult as a single large duct may be converted to multiple segmental ducts on re-resection adding to the overall morbidity post-resection. In their series, Ribero et al. reported a higher incidence of biliary fistula in those patients who underwent re-resection (44% vs 17%) [10]. However, we did not encounter increased morbidity in those patients in whom re-resection of proximal margins was performed. Another factor which may impact re-resection and margin positivity is the long length of the left hepatic duct as compared to the right which can enable re-resection of proximal margins easier and safer on the left side, thus favouring right liver resections more than left.

The impact of FS positive margins and achieving a secondary R0 is debated since a very long time. Despite its obvious theoretical advantage, there are recommendations ‘for’ and ‘against’ re-resection. Studies which do not exhibit any long-term survival advantage have recommended against re-resection [9, 14, 25]. In contrast, there are studies that found similar OS in patients with secondary R0 as compared to primary R0 which recommend the use of intraoperative FS analysis and re-resection [26, 27]. Patients who achieved secondary R0 in our study had a median survival which was significantly less than those who achieved primary R0 resection (*p* = 0.027). Similarly, no significant survival benefit was found for secondary R0 resections as compared to those patients with final R1 resections. This suggests that achieving secondary R0 margins does not translate into longer DFS or OS. In our study, the median OS of patients with initial FS margin-negative was 58.2 months as compared to 19.6 months for those with initial FS margin-positive patients (*p* = 0.012) suggesting that patients with initial FS margin-positive resections may reflect bad biology tumours with a shorter OS. These findings were described in the study conducted by Kawano et al. who found a significant dip in DFS as well as OS in patients with initial margin-positive resections on FS. Another finding in our study which reflects poor biology is the higher percentage of node-positive disease, which was found in 50% of patients with initial margin-positive tumour. Nodal involvement in pCCA is a marker of poor long-term survival and is likely a contributing factor to the poor OS and DFS in these patients with initially margin-positive tumour [28]. Thus, tumour biology not only has a negative effect on survival but also makes the therapeutic efficacy of re-resection questionable. The incidence of post-hepatectomy liver failure (PHLF) and mortality remains high after major resections for perihilar cholangiocarcinoma. A recent study by van Keulen et al. reported the incidence of liver failure and 90-day mortality as high as 20.9% and 17.0% in the 253 patients of resected pCCA, respectively [29]. Our study also reports a high 90-day mortality in 10 (28.5%). The cause of death in all these patients was PHLF precipitated by post-hepatectomy haemorrhage in 1 patient and post-hepatectomy bile leak in 2 patients. Five out of remaining 7 patients had presented with cholangitis for which biliary drainage (multiple times in some patients) was needed. Bednarsch et al. concluded that preoperative FLR < 40% and preoperative cholangitis are two risk factors for independently predicting perioperative morbidity and mortality in resected pCCA [30].

This study has several limitations. Firstly, being a retrospective study suffers from inherent bias. During the 9 years of the study period, surgical management as well as neoadjuvant/adjuvant chemotherapy for pCCA have greatly evolved. The sample size of the study was small especially after excluding postoperative 90-day mortality from the survival analysis. Our survival analysis based on this small cohort should be interpreted with caution.

## Conclusion

Intraoperative FS analysis remains a widely used test to assess the presence of cancer at the proximal cut ends of pCCA, having low sensitivity but high specificity. However, having a positive margin on FS itself portends a poor prognosis with a questionable benefit of achieving secondary R0 margins by re-resection. This undermines the utility of routine intraoperative FS for proximal margins in pCCA and its widespread applicability. Thus, considering this finding as a surrogate marker for an unfavourable disease biology, it is important to weigh the pros and cons before proceeding with additional resection.

## Data Availability

The datasets generated during and/or analysed during the current study are available from the corresponding author on reasonable request.
